# Predictive significance of hypertension in the incidence of complications in critically ill patients with COVID‑19: A retrospective cohort study

**DOI:** 10.3892/mi.2024.198

**Published:** 2024-10-09

**Authors:** Anita Katić, Nermina Rizvanović

**Affiliations:** 1 Practice of Dr Nada Vukicevic General Medicine, D-88662 Überlingen, Germany; 2 Department of Anesthesiology, Resuscitation and Intensive Care, Cantonal Hospital Zenica, University of Zenica, Faculty of Medicine, 72000 Zenica, Bosnia and Herzegovina

**Keywords:** hypertension, coronavirus disease 2019, critically ill, complications, predictive significance

## Abstract

The association between hypertension as a pre-existing comorbidity and the severe form of coronavirus disease 2019 (COVID-19) remains unclear due to the contradictory results of previously published studies. The present study evaluated the predictive significance of hypertension in the incidence of complications among critically ill patients with COVID-19. The present study included 372 critically ill adults with COVID-19 pneumonia, hospitalized between January 1 and December 31, 2021. The study cohort was divided into the hypertension group (HTA group), which included 245 patients with a history of hypertension, or a non-HTA group (control group), which included 127 patients without hypertension. The incidence of complications was retrospectively extracted from medical records and compared between groups. Multivariate regression analysis (adjusted for potential confounders) and receiver operating characteristic (ROC) curve analysis determined the predictive significance of hypertension on the incidence of complications. The patients in the HTA group were more likely to receive invasive mechanical ventilation [odds ratio (OR), 1.696; P<0.02], develop sepsis (OR, 1.807; P<0.01) and develop complications (OR, 3.101; P<0.001). Hypertension was an independent positive predictor for invasive mechanical ventilation [area under the curve (AUC), 0.67; positive predictive value (PPV), 71.7%; P<0.05], sepsis (AUC, 0.69; PPV, 77.5%; P<0.026) and total complications per patient (AUC, 0.71; PPV, 81.4%; P<0.001). On the whole, the data of the present study indicate that a history of hypertension should be considered as an independent clinical predictor of a higher incidence of complications in critically ill patients with COVID-19. Patients with pre-existing hypertension and a diagnosis of COVID-19 require timely identification, additional attention and treatment to avoid a critical course and help improve outcomes.

## Introduction

The coronavirus disease 2019 (COVID-19) pandemic, caused by severe acute respiratory syndrome coronavirus 2 (SARS-CoV-2), is considered one of the greatest global public health crises. Initial respiratory infections can rapidly progress to severe pneumonia in predisposed individuals. Of note, ~33% of patients hospitalized due to COVID-19-associated pneumonia develop acute respiratory distress syndrome (ARDS), and 16-26% of patients require intensive care unit (ICU) admission and treatment with invasive mechanical ventilation (IMV) (1). Other complications, such as sepsis, acute coronary syndrome (ACS), acute renal failure (ARF) and thromboembolic events, occur in patients undergoing treatment in the ICU. The mortality rate among critically ill patients with COVID-19 is as high as 78% (2), depending on the geographic and social characteristics of the population.

SARS-CoV-2 infection reduces the expression of angiotensin-converting enzyme II (ACE2) receptors on the surface of type II pneumocytes, and cardiac, renal, brain, intestinal and endothelial cells, leading to an imbalance in the renin-angiotensin system (RAS) (3). An overactivated angiotensin II (Ang II)-Ang II type I receptor (AT1R) axis promotes inflammatory signaling, cytokine storms, massive endothelial cell injury and coagulation disorders (4).

Previous studies have found that hypertension is one of the most common comorbidities among patients with COVID-19 (5,6). The proposed link between hypertension and COVID-19 is the interruption of the classical RAS pathway due to the downregulation of the ACE2 receptors, and hyperactivation of the Ang II-AT1R axis. Hypertension itself induces endothelial inflammation, and weakens the immune response, causing target organ damage that may increase susceptibility to serious complications of COVID-19(3). The association between hypertension and the severe form of COVID-19 remains unclear, as certain studies have reported that hypertension increases the severity or mortality from COVID-19 (2,5,7), while others have not found such evidence (8,9).

In May 2023, the World Health Organization declared the end of the COVID-19 pandemic. Epidemiologists have predicted that COVID-19 will continue with annual fluctuations in infection and may persist beyond 2025(10). Considering that an estimated 1.28 billion adults worldwide have hypertension (11), and no specific drug has yet been approved for the treatment of COVID-19, the aim of the present study was to investigate whether hypertension contributes to the development of complications in patients undergoing treatment in the ICU for COVID-19.

The present study aimed to assess the incidence of complications in critically ill patients with COVID-19-associated pneumonia and a history of hypertension, as compared with patients without hypertension. The predictive significance of hypertension in the incidence of complications in critically ill patients with COVID-19 pneumonia was determined.

## Patients and methods

### Study design

The present retrospective single-center study was conducted between January 1 and December 31, 2021, at the Department of Anesthesiology, Resuscitation, and Intensive Care of the Cantonal Hospital in Zenica, Bosnia and Herzegovina. The study report followed the Strengthening the Reporting of Observational Studies in Epidemiology (STROBE) guidelines (12).

### Ethics approval

The Research Hospital Ethics Committee of the Zenica Cantonal Hospital approved the study protocol (approval no. 00-03-35-337-23/23). The study followed the Declaration of Helsinki (13), and written informed consent was obtained from all subjects or their next-of-kin.

### Patient selection

The present study included data from 372 critically ill adults diagnosed with COVID-19. COVID-19 infection was confirmed by the reverse transcription PCR of nasopharyngeal swab samples. The criteria for admission to the ICU were as follows: Respiratory distress with dyspnea and tachypnea (>30/min), oxygen saturation in room air <90%, a ratio of partial pressure of oxygen in arterial blood to fraction of inspired oxygen (PaO_2_/FiO_2_) <300 mmHg, and the progression of pulmonary infiltrates by >50% within 48 h on a chest radiography (14). Patients with incomplete medical records, those aged <18 years, those resuscitated immediately upon admission to the ICU, those hospitalized <24 h in the ICU, or those with COVID-19 infection, but admitted to the ICU for reasons other than pneumonia (e.g., pregnancy, trauma, tumor surgery or chronic organ dysfunction) were excluded from the study. Patients who met the study criteria were divided into the hypertension group (HTA group; study group), which included 245 patients with a history of hypertension, and a non-HTA group (control group), which included 127 patients without hypertension. Hypertension was defined as an office systolic blood pressure (SBP) ≥140 mmHg and/or diastolic BP (DBP) ≥90 mmHg according to the European Society of Cardiology (ESC) and the European Society of Hypertension (ESH) (15). All patients received the same therapeutic protocol, including respiratory support, ventilation strategy, corticosteroids (methylprednisolone), anticoagulants (low molecular weight heparin), antibiotics (second generation cephalosporins), antivirals (remdesivir) and immunomodulatory therapy (tocilizumab). Patients in the HTA group continued with previously prescribed antihypertensive medications.

### Data collection

Data were retrieved from the electronic medical records by two researchers. Demographic and clinical data included age, sex, comorbidities, time from onset of symptoms to hospitalization (T1), time from hospitalization to ICU admission (T2) and the length of stay in the ICU (T3). Hemodynamic parameters recorded at ICU admission, such as SBP, DBP, mean BP (MBP) and heart rate (HR) were also collected. Initial BP was classified into grades according to the ESC and ESH in both groups. The type and number of anti-hypertensive drugs were documented in the HTA group.

### Study outcomes

The primary outcomes were intra-group differences in the incidence of the following complications: IMV, sepsis, ACS, ARF, hemodialysis, cerebrovascular insults (CVIs), thromboembolic complications, pleural abnormalities, surgical procedures and total complications per patient. The secondary aim was to determine the predictive value of hypertension in the incidence of complications in critically ill patients with COVID-19.

### Definitions of complications

IMV was defined as a therapeutic option for ARDS refractory to non-invasive respiratory support, altered consciousness and the progression of gas analysis abnormalities (pH ≤7.25, PaO_2_ ≤45 mmHg, SpO_2_ <85% with FiO_2_ 0.5). Sepsis was defined according to The Third International Consensus Definitions for Sepsis and Septic Shock. ACS was defined according to The Fourth Universal Definition of Myocardial Infarction. ARF and hemodialysis were defined according to the Kidney Disease Improving Global Outcomes Clinical Practice Guidelines for Acute Kidney Injury. CVI was defined as an acute, focal or global neurological disorder caused by a cerebral infarction or hemorrhage. Thromboembolic complications (acute limb ischemia, pulmonary embolism and intestinal infarction) were defined as thrombotic occlusions of circulation in the extremities, lungs and mesenteric/bowel blood vessels. Pleural abnormalities (pneumothorax and pneumomediastinum) were defined as the presence of air in the pleural space or mediastinum. Surgical procedures (arterial thrombectomy, amputation of ischemic limbs and laparotomy) were defined as therapeutic treatments for thromboembolic and ischemic events or gastrointestinal bleeding, and were performed under general, spinal or regional anesthesia.

### Statistical analysis

Statistical analysis was performed using SPSS v. 25.0 (IBM Corp.). The normality of the data distribution was confirmed using the Kolmogorov-Smirnov test. Categorical variables are presented as frequencies (percentages) and compared using the Pearson's χ^2^ test and Fisher's exact test. Continuous variables are presented as the mean ± standard deviation or as a median (interquartile range) and tested using an unpaired Student's t-test or Mann-Whitney U test. A multivariate logistic regression analysis (adjusted for potential confounders) was used to determine the predictive accuracy of hypertension for the incidence of complications. The predictive discriminative power of hypertension was determined using receiver operating characteristic (ROC) curve analysis. The area under the curve (AUC), specificity, sensitivity, positive predictive value (PPV) and negative predictive value were calculated. A two-sided P<0.05, 95% confidence interval and an expected study power of 80% were considered to indicate a statistically significant difference.

## Results

### Baseline demographic and clinical characteristics of the patients in the two groups

**Figure 1:**
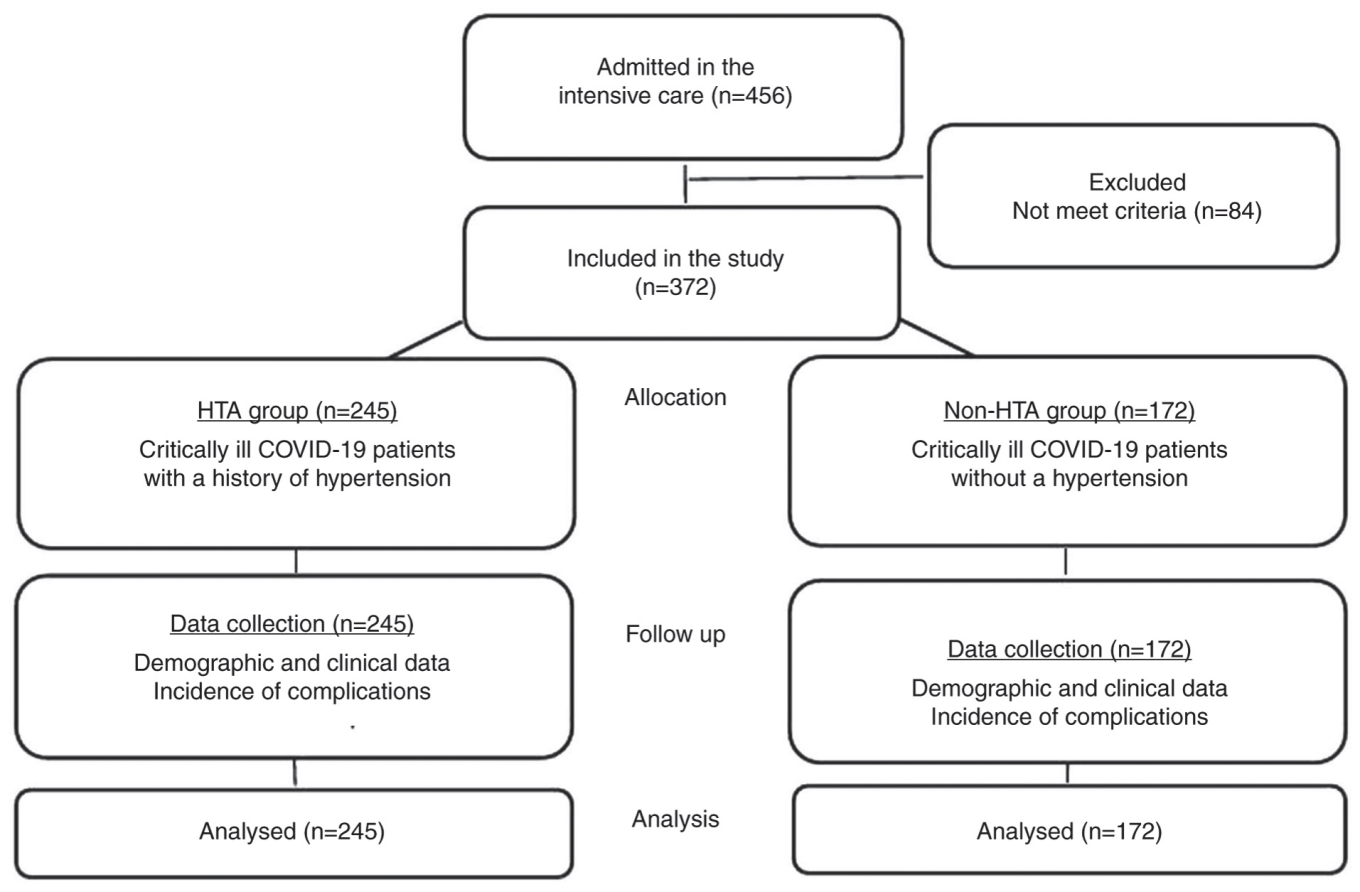
Flowchart diagram of the inclusion process of the patients in the present study.

In 2021, 456 adult patients with COVID-19 were admitted to the ICU. A total of 372 patients met the eligibility criteria, and were included in the present study and analyzed. There were 245 (65.8%) patients with hypertension and 127 (34.2%) without a history of hypertension (Fig. 1).

The median age was higher in the HTA group [67 (61.5-72.3) years] than in the control group [62 (53.1-68.5) years; P<0.001]. There were more male than female patients in both groups, although no significant difference was found. Coronary heart disease (36.3%) was the most common comorbidity in the HTA group, whereas chronic obstructive pulmonary disease (14.2%) was the most common comorbidity in the control group. The average number of comorbidities per patient was higher in the HTA group (2.14±0.97 vs. 0.94±0.34; P<0.001). Significantly higher SBP, DBP, MBP and HR values at ICU admission were observed in the HTA group (P<0.001). In the HTA group, initial BP was most frequently classified as grade 1 hypertension [80 patients (32.7%)]. In the control group, initial BP was most often classified as high-normal [42 patients (33.2%)]. ACE inhibitors were the most common anti-hypertensive therapy administered to 183 patients (74.6%) in the HTA group (Table I).

### Comparison of the incidence of complications between groups

A significantly higher incidence of IMV (P<0.001), sepsis (P<0.001), ACS (P<0.043), ARF (P<0.007), hemodialysis (P<0.018), CVIs (P<0.037), surgical procedures (P<0.09) and total complications per patient (P<0.001) was noted in the HTA group. There were 27 (11%) patients without complications in the HTA group compared with 43 (33.9%) in the control group (P<0.001; Table II).

### Predictive significance of hypertension for the incidence of complications

Univariate regression analysis revealed that hypertension had a potential predictive significance for the incidence of IMV (P<0.001), sepsis (P<0.001), ACS (P<0.040), surgical interventions (P<0.036) and total complications per patient (P<0.001; Table III).

After adjusting for age, a history of coronary heart disease and diabetes mellitus, multivariate regression analysis confirmed the independent predictive significance of hypertension on the incidence of IMV (OR=1.696; P<0.02), sepsis (OR=1.807; P<0.01) and total complications per patient (OR=3.101; P<0.001) in critically ill patients with COVID-19 (Table IV).

ROC curve analysis demonstrated the predictive discriminative power of hypertension for the incidence of IMV (AUC, 0.67; PPV, 71.7%; P<0.05), sepsis (AUC, 0.69; PPV, 77.5%; P<0.026) and total complications per patient (AUC, 0.71; PPV, 81.4%; P<0.000; Table V).

## Discussion

The present retrospective study investigated the predictive significance of pre-existing hypertension on the incidence of complications in critically ill patients with COVID-19-associated pneumonia. The results revealed that patients with a history of hypertension had a higher incidence of complications during treatment in the ICU. Hypertension was identified as an independent positive predictor of IMV, sepsis and total complications per patient. Hypertension increased the OR for IMV by 1.696-fold, sepsis by 1.807-fold and total complications per patient by 3.101-fold.

The COVID-19 pandemic has officially ended; however, the virus remains widespread worldwide. The emergence of more transmissible and virulent variants of SARS-CoV-2 will continue to pose a threat to global health (16). Patients with chronic diseases, such as hypertension, are still at risk of developing severe forms of COVID-19 and related complications. The prevalence of hypertension in patients with COVID-19 is dependent on the rate of hypertension in the general population and the form of COVID-19. In the present study, hypertension was the most prevalent comorbidity, present in 65.8% of the critically ill patients. Cummings *et al* (17) reported a 63% prevalence of hypertension in patients in the ICU.

In the present study, the median age and rates of coronary heart disease and diabetes mellitus were higher in the HTA group than those in the control group. To avoid confounding factors within baseline characteristics, the multivariate regression analysis was adjusted for age, a history of coronary heart disease and diabetes mellitus. Both groups were predominantly male, which was consistent with previous findings (7,9,18).

In the present study, in 18.9% of the patients in the control group, the mean value of BP at ICU admission was classified as grade 1 hypertension, although they had no history of hypertension. This could have been associated with COVID-19-specific mechanisms, such as cardiovascular consequences of unopposed Ang II-AT1R axis (vasoconstriction, renal salt and water retention, increased sympathetic tone), hypoxia and inflammation (3), but also non-specific mechanisms, such as corticosteroid therapy, fluid and hemodynamic management, sleep deprivation and psychosocial stress.

Despite a large body of research, the difference in complication rates between patients critically ill with COVID-19 with and without hypertension has been limited. The present study found a higher incidence of all the assessed complications in the HTA group. The most common complications were IMV, sepsis, ARF and ACS. Chen *et al* (18) reported a similar distribution of complications in hypertensive patients with COVID-19 hospitalized inside and outside the ICU, including ARDS, heart failure, sepsis and ARF.

During the COVID-19 pandemic, IMV was the main supportive treatment for hypoxemic ARDS caused by viral alveolar damage and immune-cell infiltration. The IMV rate was 29.1-94%, depending on differences in intubation criteria, ICU resources and target study populations (19,20). In the present study cohort, the incidence of IMV was 80.6%. IMV was required in 215 (87.8%) patients in the HTA group and in 88 (66.9%) patients in the control group.

COVID-19-related sepsis is of viral origin and is based on a hyperinflammatory response to viral infection and consequent immunosuppression, which induces cell apoptosis, hemodynamic instability, metabolic failure and multi-organ damage (21). Herein, sepsis rates of 57.6 and 27.6% were recorded in the HTA and control groups, respectively. Hypertension delays viral clearance, which could contribute to a higher sepsis rate in the HTA group (3).

The mechanisms involved in COVID-19-related ARF include direct viral injury to the kidneys, systemic effects of infection and secondary effects (hemodynamic failure and therapeutic consequences) (22). In the present study, ARF occurred in 50 (20.4%) patients in the HTA group, of whom 10 (20%) required hemodialysis. In the control group, ARF occurred in 12 (9.4%) patients and there was no need for hemodialysis. These findings suggested an adverse effect of pre-existing hypertension on the compensatory capacity of target organs.

The reported rate of COVID-19-related ACS is 16.1-23.8% (23). Cardiotropic viral properties and hyperinflammation cause endotheliitis, a supply/demand imbalance, and a hypercoagulable state, leading to coronary thrombosis. In the present study, the ACS rates were 17.1 and 9.4% in the HTA and control groups, respectively. Hypertension may worsen the rate of ACS with chronically increased sympathetic activity, arterial stiffness and impaired myocardial relaxation (3).

Despite routine thromboprophylaxis, thromboembolic events were recorded in 10.6 and 4.7% of the patients in the HTA and control groups, respectively. Klok *et al* (24) reported a 31% incidence of thrombotic complications in patients with COVID-19in the ICU. The discrepancy in the results between the studies could be explained by the different classifications of complications.

In the literature, the incidence of CVI (ischemic and hemorrhagic) in patients critically ill with COVID-19 was found to be up to 6% (25). In the present study, the rate of CVI in the HTA group was 3.3% and there were no CVIs in the control group. Such a low rate of CVIs was probably underestimated, due to the difficulty of neurological assessment in intubated and sedated patients.

A higher rate of pleural abnormalities was noted in the HTA group due to a greater need for IMV; however, there was no statistically significant difference between the groups.

As was expected, surgical procedures were performed more often in the HTA group due to thromboembolic complications, and acute ischemic and hemorrhagic events were more common in this group.

Previous studies have evaluated the impact of a history of hypertension on the severity of COVID-19, although with very inconsistent results, due to large heterogeneity across the studies, including patient populations (7,9,18), disease course (7,9,18), the definition of hypertension (8,18,26), statistical analysis (2,9,26,27,28) and study outcomes (29,30). In order to minimize bias, the present study focused exclusively on complications in critically ill patients with COVID-19 and pre-existing hypertension, and included multivariate regression analysis adjusted for potential confounders. These results provided evidence that a history of hypertension increased the incidence of complications in critically ill patients with COVID-19, and revealed hypertension as a predictor of IMV, sepsis and total complications per patient. There lies the strength of the present study. The study limitations include its retrospective, single-center design and the absence of external validation. The present study also did not evaluate ICU mortality rates or follow-up of patients following ICU and hospital discharge.

In conclusion, pre-existing hypertension predicts a higher incidence of complications in critically ill patients with COVID-19. Early identification and effective treatment of COVID-19 patients with a history of hypertension are paramount to avoid disease progression and complications.

## Figures and Tables

**Table I:** Baseline demographic and clinical characteristics of the study participants.

Parameters	HTA group, n=245 (65.8%)	non-HTA group, n=127 (34.2%)	P-value
Age, years (median, IQR)	67 (61.5-72.3)	62 (53.1-68.5)	**0.001** ^a^
Sex, n (%)			0.067^b^
Male	141 (57.6)	86 (67.7)	
Female	104 (42.4)	41 (32.3)	
Comorbidities, *n* (%)			
Coronary heart disease			**0.001** ^b^
Yes	89 (36.3)	12 (9.4)	
No	156 (63.7)	115 (90.6)	
Diabetes mellitus			**0.001** ^b^
Yes	81 (33.1)	17 (13.4)	
No	164 (66.9)	110 (86.6)	
COPD			0.162^b^
Yes	23 (9.4)	18 (14.2)	
No	222 (90.6)	109 (85.8)	
Hypothyroidism			0.096^b^
Yes	21 (8.6)	5 (3.9)	
No	224 (91.4)	122 (96.1)	
Chronic kidney disease			0.174^c^
Yes	9 (3.7)	1 (0.8)	
No	236 (96.3)	126 (99.2)	
Cerebrovascular disease			0.171^b^
Yes	8 (3.6)	8 (6.3)	
No	237 (96.4)	199 (93.6)	
Average of comorbidities			
per patient (mean ± SD)	2.14±0.97	0.94±0.34	**0.001** ^d^
T1, days (median, IQR)	7.0 (5.0-10.0)	7.0 (5.0-9.0)	0.121^a^
T2, days (median, IQR)	3.0 (1.0-5.0)	3.0 (2.0-5.0)	0.070^a^
T3, days (median, IQR)	6.0 (4.0-8.5)	7.0 (4.0-9.0)	0.346^a^
SBP, mmHg (mean ± SD)	145.04±17.15	132.28±7.86	**0.001** ^d^
DBP, mmHg (mean ± SD)	106.97±15.48	96.19±10.46	**0.001** ^d^
MBP, mmHg (mean ± SD)	88.04±12.85	79.48±9.81	**0.001** ^d^
Heart rate, beat/min (mean ± SD)	101.88±21.19	91.96±15.88	**0.001** ^d^
Hypertension grade, n (%)			**0.001** ^b^
Optimal BP, <120 mmHg	26 (10.6)	25 (19.6)	
Normal BP, 120-130/80-84 mmHg	29 (11.8)	36 (28.3)	
High-normal BP, 130-139/85-89 mmHg	43 (17.6)	42 (33.2)	
Grade 1 HTA, 140-159/90-99 mmHg	80 (32.7)	24 (18.9)	
Grade 2 HTA, 160-179/100-109 mmHg	43 (17.6)	/	/
Grade 3 HTA, >180/110 mmHg	24 (9.8)	/	/
Type of anti-hypertensive drugs, n (%)			
ACE inhibitors	183 (74.6)	/	/
Angiotensin receptor blockers	31 (12.7)	/	/
Calcium channel blockers	67 (27.3)	/	/
β-blockers	112 (45.7)	/	/
Diuretics	100 (40.8)	/	/
Average of antihypertensive drugs			
per patient (mean ± SD)	2.70±1.34	/	/

Values in bold font indicate statistically significant differences (P<0.05). P-values were obtained using: the

^a^Mann-Whitney U test,

^b^Pearson's χ^2^ test,

^c^Fisher's exact test,

^d^Student's t-test. HTA group, patients with hypertension; non-HTA group, patients without hypertension; IQR, interquartile range; COPD, chronic obstructive pulmonary disease; SD, standard deviation; T1, time from onset of symptoms to hospitalization; T2, time from hospitalization to intensive care unit admission; T3, length of stay in the intensive care unit; SBP, systolic blood pressure; DBP, diastolic blood pressure; MBP, mean blood pressure; ACE, angiotensin convertase enzyme.

**Table II:** Incidence and distribution of complications according to the groups.

Complications	HTA group, n=245 (65.8%)	non-HTA group, n=127 (34.2%)	95 % CI	P-value
IMV, n (%)			0.00-0.00	**0.001** ^a^
Yes	215 (87.8)	85 (66.9)		
No	30 (12.2)	42 (33.1)		
Sepsis, n (%)			0.00-0.00	**0.001** ^a^
Yes	141 (57.6)	35 (27.6)		
No	34 (27.6)	92 (72.4)		
Acute coronary syndrome, n (%)			0.05-0.06	**0.043** ^a^
Yes	42 (17.1)	12 (9.4)		
No	203 (82.9)	115 (90.6)		
Acute renal failure, n (%)			0.00-0.01	0.007^a^
Yes	50 (20.4)	12 (9.4)		
No	195 (79.6)	115 (90.6)		
Hemodialysis, n (%)			0.02-0.03	**0.018** ^b^
Yes	10 (4.1)	0 (0)		
No	235 (95.9)	127(100)		
Cerebrovascular insults, n (%)			0.04-0.05	**0.037** ^b^
Yes	8 (3.3)	0 (0)		
No	237 (96.7)	127(100)		
Thromboembolic events, n (%)			0.07-0.08	0.055^a^
Yes	26 (10.6)	6 (4.7)		
No	219 (89.4)	121 (55.3)		
Pleural abnormalities, n (%)			0.12-0.13	0.131^b^
Yes	15 (6.1)	3 (2.4)		
No	230 (93.9)	124 (97.6)		
Surgical procedures, n (%)			0.01-0.01	**0.009** ^b^
Yes	14 (4.6)	1 (0.8)		
No	231 (95.4)	126 (99.2)		
Average of complications				
per patient, (mean ± SD)	2.11±1.1	1.2±0.7	0.00-0.00	**0.001** ^c^
Distribution of complications				**0.010** ^b^
No complications, n (%)	27 (11.0)	43 (33.9)	0.00-0.00	
1 complication, n (%)	43 (17.6)	34 (2.8)	0.04-0.04	
2 complications, n (%)	76.(31.1)	31 (24.4)	0.18-0.19	
3 complications, n (%)	75 (30.6)	15 (11.8)	0.00-0.00	
4 complications, n (%)	19 (7.8)	3 (2.4)	0.03-0.04	
5 complications, n (%)	5 (2.0)	0 (0.0)	0.16-0.17	

Values in bold font indicate statistically significant differences (P<0.05). P-values were obtained using the:

^a^Pearson's χ^2^ test,

^b^Fisher's exact test,

^c^Student's t-test. HTA group, patients with hypertension; non-HTA group, patients without hypertension; CI, confidence interval; IMV, invasive mechanical ventilation; SD, standard deviation.

**Table III:** Univariate regression analysis.

	Univariate regression analysis		
Complications	OR	95% CI	P-value
IMV	3.541	2.08-6.02	**0.001**
Sepsis	3.564	2.24-5.67	**0.001**
Acute coronary syndrome	1.983	1.00-3.91	**0.040**
Acute renal failure	2.457	1.25-4.80	0.009
Hemodialysis	8.730	0.00-0.00	0.999
Cerebrovascular insults	8.656	0.00-0.00	0.999
Thromboembolic events	2.394	0.95-5.97	0.061
Pleural abnormalities	2.696	0.76-9.49	0.123
Surgical procedures	8.803	1.15-6.71	**0.036**
Total complications per patients	1.987	1.61-2.44	**0.001**

Values in bold font indicate statistically significant differences (P<0.05). OR, odds ratio; CI, confidence interval; IMV, invasive mechanical ventilation.

**Table IV:** Multivariate regression analysis.

	Multivariate analysis					
	Unadjusted model			Adjusted model^a^		
Complications	OR	95% CI	P-value	OR	95% CI	P-value
IMV	2.076	0.64-4.17	**0.001**	1.696	0.92-2.91	**0.02**
Sepsis	2.416	0.59-4.36	**0.001**	1.807	0.97-3.58	**0.01**
Acute coronary syndrome	0.537	0.20-1.41	0.208	/	/	/
Acute renal failure	0.443	0.16-1.18	0.104	/	/	/
Surgical procedures	0.512	0.19-1.37	0.193	/	/	/
Total complications per patients	4.025	0.91-7.99	**0.001**	3.101	1.57-6.10	**0.001**

Values in bold font indicate statistically significant differences (P<0.05).

^a^Adjusted model, predictive significance of hypertension on the incidence of complications after adjustment for age, history of coronary heart disease and diabetes mellitus. OR, odds ratio of the predictor variable; CI, confidence interval; IMV, invasive mechanical ventilation

**Table V:** Predictive discriminatory power of hypertension on the incidence of complications.

	IMV	Sepsis	Total complications per patient
AUC	0.67	0.69	0.71
Sensitivity %	67.1	69.6	73.4
Specificity %	60.9	64.0	63.2
PPV %	71.7	77.5	81.4
NPV %	34.5	37.7	50.1
95% CI	0.48-0.70	0.54-0.79	0.65-0.81
P-value	**0.05**	**0.026**	**0.001**

Values in bold font indicate statistically significant differences (P<0.05). P-values were obtained using receiver operating characteristic (ROC) curve analysis. IMV, invasive mechanical ventilation; AUC, area under curve; PPV, positive predictive value; NPV, negative predictive value; CI, confidence interval.

## Data Availability

The datasets used and/or analyzed during the current study are available from the corresponding author on reasonable request.
